# Protective Effect of Ganjang, a Traditional Fermented Soy Sauce, on Colitis-Associated Colorectal Cancer in Mice

**DOI:** 10.3390/foods14040632

**Published:** 2025-02-13

**Authors:** Hyeon-Ji Lim, In-Sun Park, Min Ju Kim, Ji Won Seo, Gwangsu Ha, Hee-Jong Yang, Do-Youn Jeong, Seon-Young Kim, Chan-Hun Jung

**Affiliations:** 1 Jeonju AgroBio-Materials Institute, Wonjangdong-gil 111-27, Jeonju 54810, Republic of Korea; lhj0923@jami.re.kr (H.-J.L.); witwit58@jami.re.kr (I.-S.P.); mjkim92@jami.re.kr (M.J.K.); seon02@jami.re.kr (S.-Y.K.); 2 Microbial Institute for Fermentation Industry, Sunchang 56048, Republic of Korea; wldnjs8769@naver.com (J.W.S.); ksnova1492@naver.com (G.H.); godfiltss@naver.com (H.-J.Y.); jdy2534@korea.kr (D.-Y.J.)

**Keywords:** colorectal cancer, diet, microbial factors, ganjang, fermented soy sauce, functional food

## Abstract

Colorectal cancer (CRC) is one of the most prevalent causes of cancer-related fatalities globally, and its development is closely associated with dietary and microbial factors. The aim of this study was to investigate the potential of ganjang, a traditional fermented soy sauce, in alleviating colitis-associated colorectal cancer (CAC) in a mouse model induced by azoxymethane/dextran sulfate sodium (AOM/DSS). The bacterial composition of ganjang samples from different regions primarily included *Lactobacillus* spp. and *Bacillus* spp. Administration of ganjang to AOM/DSS-induced mice significantly improved CAC-related symptoms, including increased body weight, restored colon length, and reduced spleen size. Additionally, ganjang administration led to a decrease in tumor size and number, the modulation of apoptotic and proliferative markers, decreased inflammatory cytokine levels, and the restoration of the intestinal epithelial barrier. Furthermore, ganjang samples altered the gut microbiota composition by increasing the relative abundance of *Lactobacillus* spp. These findings suggest that ganjang has potential as a functional food for CRC prevention or mitigation, primarily through the modulation of CAC symptoms, tumor growth, inflammatory responses, intestinal epithelial barrier integrity, and gut microbiota composition.

## 1. Introduction

Colorectal cancer (CRC) is one of the leading causes of mortality worldwide and poses a substantial public health concern [1]. Although the exact cause of CRC remains unknown despite extensive research, recent studies have highlighted diet as a major contributing factor [2]. The human gut harbors a diverse community of microorganisms, collectively referred to as the gut microbiota [3]. Gut microbes play an essential role in maintaining health by facilitating digestion, modulating immune responses, and regulating metabolic processes [4,5]. Studies have shown that dysbiosis, characterized by imbalances in microbial diversity, with an overrepresentation of pathogenic bacteria and a reduction in beneficial microbes, is associated with an increased risk of CRC [6]. Dietary components, particularly the probiotics found in fermented foods, have been shown to modulate gut microbiota composition, promote antitumor immunity, and potentially reduce the risk of cancer development [7]. Probiotics, such as *Lactobacillus* spp., commonly found in fermented products, help alleviate various diseases such as inflammatory bowel disease and CRC by enhancing gut barrier function, suppressing inflammation, and promoting immune homeostasis [8]. Prebiotics, defined as substrates that are selectively utilized by host microorganisms conferring a health benefit, such as fructooligosaccharides (FOSs) and isomalto-oligosaccharides (IMOs), also promote the growth of beneficial bacteria and enhance metabolic activities in the large intestine [8].

Ganjang, a traditional fermented soy sauce integral to Korean cuisine, is widely used as a seasoning to enhance the flavor and aroma of various dishes [9], including doenjang jjigae, galbijjim, bulgogi, and other globally recognized Korean foods. Ganjang is produced by fermenting meju (boiled soybean bricks) with cheonilyeom (solar sea salt) and aging the mixture in earthenware jars [9]. Traditionally, ganjang is produced using indigenous microorganisms in natural fermentation conditions; nevertheless, it is also produced through industrial methods that employ selected microbial strains under controlled conditions for large-scale production [10]. However, the natural fermentation process in traditionally prepared ganjang provides superior flavor and taste compared to those produced using commercial methods, which could be attributed to the indigenous microbial diversity in traditional ganjang [11,12]. Ganjang is a rich source of bioactive compounds, including peptides, free amino acids (glutamate, alanine, lysine), organic acids (lactate, acetate), and polyphenols. These compounds are produced during fermentation and impart the unique flavor and aroma of ganjang [9]. In addition, these compounds contribute to the various biological activities and health benefits of ganjang. Preliminary studies suggest that ganjang may exert anti-inflammatory, antihypertensive, anti-obesity, and antioxidant effects in animal models, such as mice with dextran sodium sulfate (DSS)-induced colitis [13,14,15,16]; however, further research is needed to confirm these findings. Furthermore, the microbial composition of ganjang, which includes *Lactobacillus* spp., *Bacillus* spp., and other beneficial microbes, plays a key role in enhancing its functional properties and health benefits [9,17].

In the present study, we explored the bacterial composition of ganjang from different regions and evaluated its protective effects against CRC using a mouse model of azoxymethane (AOM)/DSS-induced colitis-associated CRC (CAC).

## 2. Materials and Methods

### 2.1. Information on Ganjang

Four types of ganjang products were obtained from the Microbial Institute for Fermentation Industry (Sunchang-gun, Jeollabuk-do, Republic of Korea). The details of the ganjang samples are listed in Table 1.

### 2.2. Animal Experiments

Thirty-five 4-week-old male BALB/c mice (18–20 g) were purchased from Damool Science (Daejeon, Republic of Korea). Mice were maintained in a specific pathogen-free facility under controlled conditions (temperature, 22 ± 2 °C; humidity, 55% ± 5%; 12 h light/dark cycle) with access to a standard diet and distilled water. The body weights of the mice were recorded two times per week. All experimental procedures were performed according to the guidelines and approved by the Animal Care and Use Committee of the Jeonju AgroBio-Materials Institute (approval number: JAMI IACUC 2023007) on 28 March 2023.

### 2.3. Animal Grouping and Introduction of Colitis-Associated Colorectal Cancer (CAC)

The mice were divided into seven groups with five mice in each group: (1) normal control (Nor), (2) AOM/DSS (A/D), (3) positive control (PC), (4) GJ1, (5) GJ2, (6) GJ3, and (7) GJ4. Mice in the Nor group received only water, while the A/D group received 10 mg/kg AOM + 2% DSS with water. The PC group was treated with AOM/DSS and 75 mg/kg/day of 5-aminosalicylic acid (5-ASA). Mice in the GJ1, GJ2, GJ3, and GJ4 groups received AOM/DSS along with 2% brine of traditionally produced ganjang samples 1, 2, and 3, and industrially produced ganjang samples, respectively. In all GJ groups (GJ1–GJ4), mice were intraperitoneally injected with 10 mg/kg AOM after administering Ganjang samples and 5-ASA orally for 8 days. Seven days later, 2% (*w*/*v*) DSS was supplied in drinking water for 7 days, followed by regular water for 2 weeks. This cycle was repeated twice.

### 2.4. Bacterial Community Analysis Using Next-Generation Sequencing (NGS)

Microbiome analysis was performed following the Illumina 16S Metagenomic Sequencing Library Preparation protocol (Part # 1544223 Rev. B). Bacterial DNA was extracted from the collected samples (ganjang and fecal samples of mice) using the DNeasy PowerSoil Pro Kit (Qiagen, Hilden, Germany). The V3–V4 regions of the 16S rRNA gene were amplified using region-specific primers. The resulting amplicons were purified using a bead-based method to remove impurities while retaining the target amplicons and desired fragment size. Sample indexing for differentiation was performed using the Nextera XT DNA Library Prep Kit (Illumina, San Diego, CA, USA). Following indexing, an additional bead-based purification step was performed to eliminate residual contaminants. Sequencing was performed on the Illumina Miseq platform, using 300 bp paired-end reads, at the Microbial Institute for Fermentation Industry (Sunchang, Republic of Korea). Raw sequencing data in FASTQ format were analyzed using the EzBioCloud 16S-based Microbiome Taxonomic Profiling (MTP) platform (Chunlab Inc., Seoul, Republic of Korea). Taxonomic assignment was performed using the EzTaxon database PKSSU version 4.0 [18]. The sequences were classified taxonomically at multiple hierarchical levels, including phylum, class, order, family, genus, and species. Within-sample diversity was assessed by calculating α-diversity indices (Chao and Shannon) using Mothur (v.1.36.).

### 2.5. Determination of Inflammatory Cytokines in the Serum

Serum TNF-α, IL-1β, and IL-6 levels were measured using enzyme-linked immunosorbent assay (ELISA) (R&D system, Minneapolis, MN, USA) according to the manufacturer’s instructions.

### 2.6. Hematoxylin–Eosin (HE) Staining

Colon tissues were fixed in 10% formalin solution, embedded in paraffin blocks, and sliced into approximately 4 µm sections. Subsequently, the sliced sections were deparaffinized and rehydrated using a xylene–ethanol–water gradient system. HE staining was performed, followed by dehydration. Histopathological analysis was performed using a digital scanner (Motic, Xiamen, China).

### 2.7. Western Blot (WB) Analysis

Colon tissue proteins were extracted using ice-cold RIPA lysis buffer (Thermo Fisher Scientific, Inc., Wilmington, MA, USA) containing 1% protease and phosphatase inhibitor cocktails (Gendepot, Barker, TX, USA). Protein concentration was quantified using BCA reagent (Bio-Rad, Hercules, CA, USA). Subsequently, equal amounts of proteins were separated using 7.5–12% SDS-PAGE (Bio-Rad), and the proteins were transferred to a PVDF membrane (Bio-Rad). The membranes were blocked with 5% skim milk for 1 h at room temperature and incubated with primary antibodies at 4 °C overnight. The membrane was then incubated for 1 h with HRP-conjugated secondary antibodies. The protein bands were visualized using ECL luminescent reagent (GE Healthcare, Chicago, IL, USA) on an Amersham Imager 600 system (GE Healthcare).

### 2.8. Quantitative Real-Time PCR (qRT-PCR) Analysis

Total RNA was extracted using an RNA purification kit (GeanAll, Seoul, South Korea) following the manufacturer’s instructions. cDNA was synthesized using a reverse transcription (RT) premix kit (BioFact, Daejeon, Republic of Korea). Subsequently, qRT-PCR amplification was performed using 2×SYBR Green qPCR Master Mix (BioFact). Finally, the relative mRNA expression levels of the genes were calculated using the 2^−ΔΔCT^ method. Primer sequences are listed in Table 2.

### 2.9. Immunohistochemistry (IHC) Assay

Paraffin-embedded colon sections (4 μm thick) were deparaffinized with xylene three times for 7 min each. The sections were then rehydrated through a series of ethanol washes, followed by washing with water, and incubated with 0.3% H_2_O_2_ for 15 min to block endogenous peroxidase activity. Antigen retrieval was performed by heating the sections in 0.01 M citrate buffer (pH 6.0) in a microwave for 15 min. Next, the tissue sections were pre-blocked with 4% bovine serum albumin for 30 min and incubated overnight with the following primary antibodies: Ki67 (Danvers, MA, USA, Cell Signaling Technology), MUC-2 (Cambridge, UK, Abcam), and TFF-3 (Abcam) at 4 °C. The sections were then treated with an anti-rabbit HRP-conjugated secondary antibody (Dako, Glostrup, Denmark), stained with hematoxylin, and analyzed using a digital scanner (Motic).

### 2.10. Statistical Analysis

All statistical analyses were performed using GraphPad Prism (version 5.0; GraphPad Software, Inc., San Diego, CA, USA) with Tukey’s post-hoc tests. *p*-values <0.05 were considered statistically significant. The results are presented as mean ± standard deviation (SD).

## 3. Results

### 3.1. Bacteria Composition in Ganjang Samples

**Figure 1:**
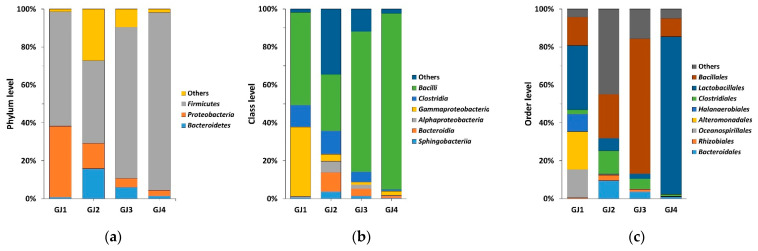

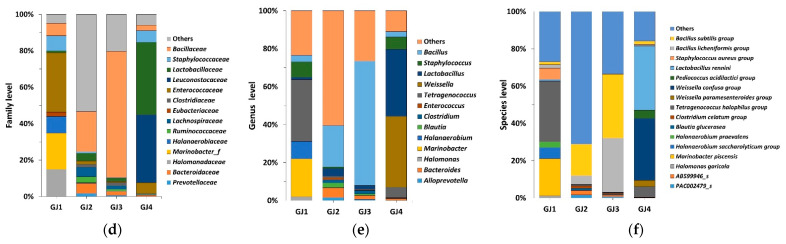
Microbial community composition in ganjang samples at the (**a**) phylum, (**b**) class, (**c**) order, (**d**) family, (**e**) genus, and (**f**) species levels assessed using next-generation sequencing analysis.

NGS analysis of genomic DNA extracted from the four ganjang samples revealed a predominance of Firmicutes and Bacilli in all samples at the phylum and class levels, respectively (Figure 1a,b). In the GJ1 samples, Lactobacillales (33.87%) were most predominant, followed by Alteromonadales (19.94%), Bacillales (15.01%), and Oceanospirillales (19.93%). In the GJ2 samples, Bacillales (23.21%) was the most predominant order, followed by Clostridiales (12.31%), Bacteroidales (9.57%), and Lactobacillales (6.60%). In the GJ3 and GJ4 samples, Bacillales (71.19%) and Lactobacillales (83.23%) were the most predominant orders, respectively (Figure 1c). At the family level, *Enterococcaceae* (32.60%) was most predominant in GJ1 samples, followed by *Marinobacter_f* (19.81%), *Halomonadaceae* (14.93%), and *Halanaerobiaceae* (9.23%). In GJ2 and GJ3, *Bacillaceae* (22.03%) was most predominant, whereas in GJ4, *Lactobacillaceae* (39.78%) and *Leuconostocaceae* (37.34%) were predominant (Figure 1d). At the genus level, *Tetragenococcus* (32.57%) was most predominant, followed by *Marinobacter* (19.82%), *Halanaerobium* (9.23%), *Staphylococcus* (8.17%), and *Bacillus* (3.53%) in the GJ1 samples. In GJ2, *Bacillus* (21.92%) was the predominant genus, followed by *Lactobacillus* (4.16%), *Bacteroides* (4.91%), *Blautia* (2.47%), and *Alloprevotella* (1.64%). In GJ3, the abundance of *Bacillus* (65.49%) was the highest, whereas GJ4 was dominated by *Weissella* (37.25%) and Lactobacillales (35.38%) (Figure 1e). At the species level, the relative abundance of *Tetragenococcus halophilus* (32.51%) was the highest in the GJ1 samples, followed by that of *Marinobacter piscensis* (19.79%), *Staphylococcus aureus* (6.03%), and *Halanaerobium saccharolyticum* (6.03%). In GJ2, *Bacillus subtilis* (16.90%) was most predominant, whereas GJ3 was dominated by *Bacillus subtilis* (34.39%) and *Bacillus licheniformis* (29.07%). In GJ4 samples, *Lactobacillus rennini* (34.46%) and *Weissella confusa* (33.29%) were predominant (Figure 1f).

### 3.2. Effect of Ganjang on Symptoms in AOM/DSS-Induced CAC Mice

**Figure 2:**
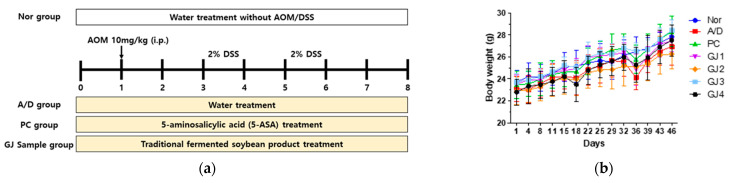

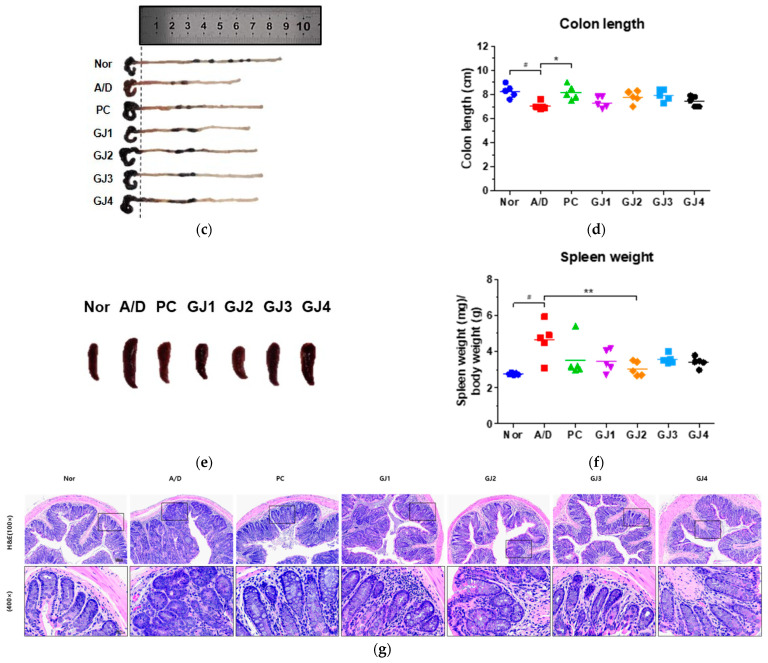
Effect of ganjang on symptoms of AOM/DSS-induced CAC mice: (**a**) Experimental procedure; (**b**) Body weight changes (**g**); (**c**) Representative images of colon; (**d**) Colon length (cm); (**e**) Representative images of spleen; (**f**) Spleen weight (mg)/body weight (g); (**g**) Representative images of HE staining on colorectal tissues (scale bar: 100 µm and 300 µm, 100× and 400× magnification). Nor, Normal control group; A/D, 10 mg/kg AOM + 2% DSS; PC, 10 mg/kg AOM + 2% DSS + 75 mg/kg 5-ASA; GJ1, 10 mg/kg AOM + 2% DSS + Ganjang sample 1; GJ2, 10 mg/kg AOM + 2% DSS + Ganjang sample 2; GJ3, 10 mg/kg AOM + 2% DSS + Ganjang sample 3; GJ4, 10 mg/kg AOM + 2% DSS + Ganjang sample 4. Data are expressed as mean ± SEM (n = 5). ^#^ *p* < 0.05 vs. Nor group; * *p* < 0.05, ** *p* < 0.01 vs. A/D group.

The effects of ganjang on CAC-related symptoms were assessed in an AOM/DSS-induced CAC mouse model. Mice received intraperitoneal injections of carcinogen AOM (10 mg/kg), followed by two cycles of 2% DSS to induce intestinal inflammation, and a subsequent 14-day recovery period (Figure 2a). No significant difference in body weight was observed in any group throughout the experimental period. However, treatment with 2% DSS decreased the body weight in the A/D group compared to that in the Nor group, which tended to be partially restored by administering ganjang samples (Figure 2b). The colon length of mice in the A/D group was significantly reduced (7.1 ± 0.3 cm) compared to that in the Nor group (8.3 ± 0.5 cm), which was observed to be slightly recovered by the administration of ganjang samples (GJ1; 7.3 ± 0.5 cm, GJ2; 7.8 ± 0.5 cm, GJ3; 7.9 ± 0.5 cm, GJ4; 7.4 ± 0.4 cm) (Figure 2c,d). Furthermore, the enlarged spleen in the A/D group (4.6 ± 1.0 mg/g) was also slightly recovered by the administration of ganjang samples (GJ1; 3.5 ± 0.6 mg/g, GJ2; 3.0 ± 0.4 mg/g, GJ3; 3.6 ± 0.3 mg/g, GJ4; 3.4 ± 0.3 mg/g) (Figure 2e,f). HE staining of colorectal tissues revealed histological abnormalities, including crypt destruction, architectural and cytological atypia, and extensive inflammatory cell infiltration, in the A/D group. These changes were markedly recovered by the administration of ganjang samples (Figure 2g).

### 3.3. Effect of Ganjang on Tumor Formation and Growth in AOM/DSS-Induced CAC Mice

**Figure 3:**
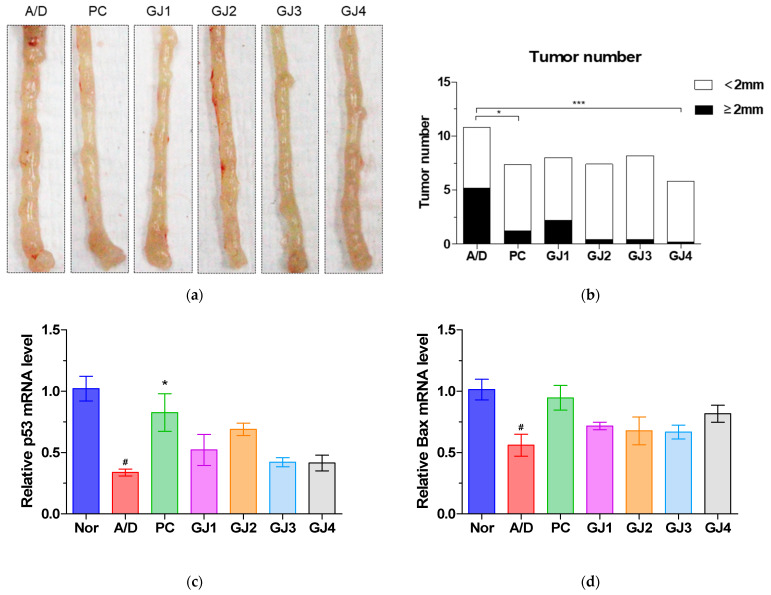

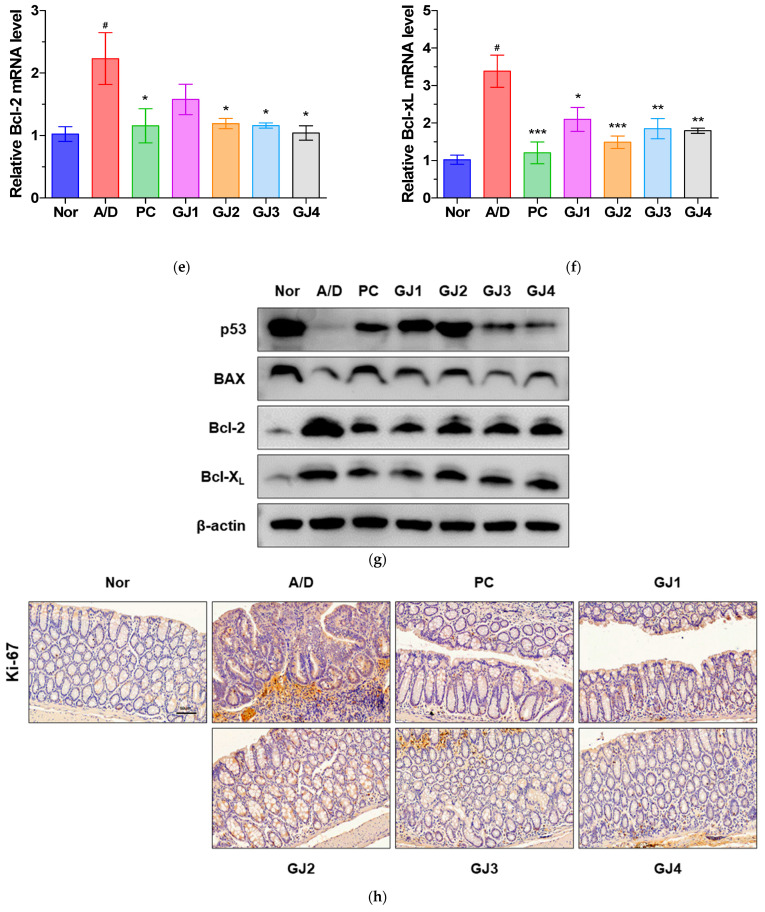
Effects of ganjang on tumor formation and growth in AOM/DSS-induced CAC mice: (**a**) Representative picture of colon tumor; (**b**) Tumor size and tumor number; (**c**–**f**) mRNA levels of p53 (**c**), Bax (**d**), Bcl-2 (**e**), and Bcl-X_L_ (**f**); (**g**) Protein levels of p53, Bax, Bcl-2, and Bcl-X_L_; (**h**) Immunohistochemical staining of Ki-67 in colon tissues (scale bar: 60 µm, 200x magnification). Nor, Normal control group; A/D, 10 mg/kg AOM + 2% DSS; PC, 10 mg/kg AOM + 2% DSS + 75 mg/kg 5-ASA; GJ1, 10 mg/kg AOM + 2% DSS + Ganjang sample 1; GJ2, 10 mg/kg AOM + 2% DSS + Ganjang sample 2; GJ3, 10 mg/kg AOM + 2% DSS + Ganjang sample 3; GJ4, 10 mg/kg AOM + 2% DSS + Ganjang sample 4. Data are expressed as mean ± SEM (n = 5). # *p* < 0.05, compared to the Nor group; * *p* < 0.05, ** *p* < 0.01, and *** *p* < 0.001, compared to the A/D group.

Tumor number and size in colorectal tissues collected from sacrificed mice (Figure 3a) were assessed using a digital caliper to investigate the effect of ganjang on tumor formation and growth. The total number of tumors increased significantly in the A/D group compared to that in the Nor group, with a similar proportion of tumors measuring ≥2 mm and <2 mm in diameter. Although the number of small tumors with a diameter of <2 mm was similar in the A/D and GJ groups, the number of large tumors (≥2 mm) was significantly reduced in the GJ groups (Figure 3b).

Next, we evaluated markers of apoptosis and cell proliferation to explore the underlying mechanisms of tumor growth inhibition. The mRNA levels of pro-apoptotic markers—p53 and Bax—in the A/D group were significantly reduced compared to those in the normal group. This effect was restored in the GJ groups (Figure 3c,d). In contrast, the mRNA levels of anti-apoptotic markers—Bcl-2 and Bcl-X_L_—significantly increased in the A/D group compared to those in the normal group, which was also restored in the GJ groups (Figure 3e,f). WB analysis revealed consistent results for the expression of p53, Bax, Bcl-2, and Bcl-X_L_ (Figure 3g). Furthermore, immunohistochemical staining to assess the expression of the cell proliferation marker Ki-67 revealed increased expression in the A/D group compared to that in the normal group. Nevertheless, ganjang administration restored the AOM/DSS-induced effects in all GJ groups (Figure 3h).

### 3.4. Effect of Ganjang on Inflammatory Cytokines in AOM/DSS-Induced CAC Mice

**Figure 4:**
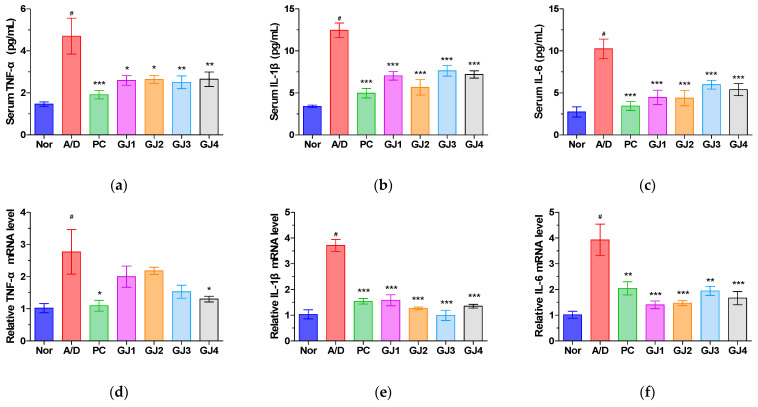
Effects of ganjang on inflammatory cytokines in AOM/DSS-induced CAC mice: (**a**–**c**) Serum levels of TNF-α (**a**), IL-1β (**b**), and IL-6 (**c**); (**d**–**f**) Protein levels of TNF-α (**d**), IL-1β (**e**), and IL-6 (**f**) in the colon tissue. Nor, Normal control group; A/D, 10 mg/kg AOM + 2% DSS; PC, 10 mg/kg AOM + 2% DSS + 75 mg/kg 5-ASA; GJ1, 10 mg/kg AOM + 2% DSS + Ganjang sample 1; GJ2, 10 mg/kg AOM + 2% DSS + Ganjang sample 2; GJ3, 10 mg/kg AOM + 2% DSS + Ganjang sample 3; GJ4, 10 mg/kg AOM + 2% DSS + Ganjang sample 4. Data are expressed as mean ± SEM (n = 5). # *p* < 0.05, vs. the Nor group; * *p* < 0.05, ** *p* < 0.01, and *** *p* < 0.001, vs. A/D group.

The effect of ganjang on inflammatory cytokine expression in AOM/DSS-induced mice was assessed using ELISA and real-time PCR. The serum levels of TNF-α, IL-1β, and IL-6 were significantly elevated in the A/D group compared to those in the normal group. Nevertheless, ganjang administration restored these increases (Figure 4a–c). Similarly, the mRNA levels of TNF-α, IL-1β, and IL-6 in colon tissues were significantly higher in the A/D group than that in the normal group; this elevation was reduced in the GJ groups (Figure 4d–f).

### 3.5. Effect of Ganjang on Intestinal Epithelial Barrier in AOM/DSS-Induced CAC Mice

**Figure 5:**
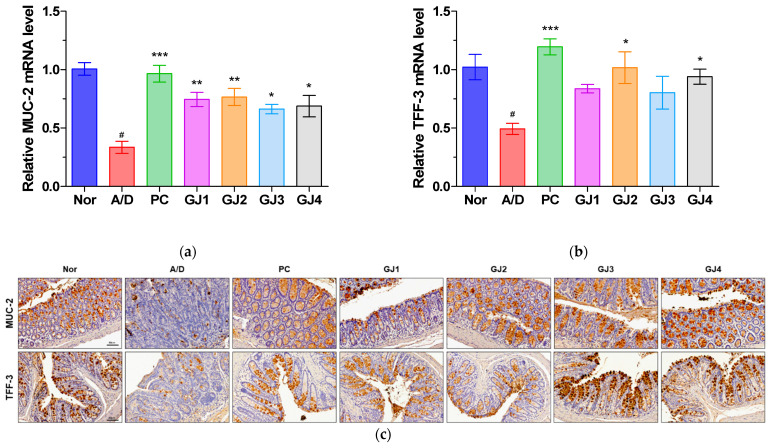
Effects of ganjang on intestinal epithelial surface in AOM/DSS-induced CAC mice. (**a**,**b**) mRNA levels of *Muc2* (**a**) and *Tff3* (**b**) in colon tissue. (**c**) Representative immunohistochemical staining images of MUC2 and TFF3 expression in colon tissue (scale bar: 60 µm, 200x magnification). Nor, Normal control group; A/D, 10 mg/kg AOM + 2% DSS; PC, 10 mg/kg AOM + 2% DSS + 75 mg/kg 5-ASA; GJ1, 10 mg/kg AOM + 2% DSS + Ganjang sample 1; GJ2, 10 mg/kg AOM + 2% DSS + Ganjang sample 2; GJ3, 10 mg/kg AOM + 2% DSS + Ganjang sample 3; GJ4, 10 mg/kg AOM + 2% DSS + Ganjang sample 4. Data are expressed as mean ± SEM (n = 5). # *p* < 0.05, compared to the Nor group; * *p* < 0.05, ** *p* < 0.01, and *** *p* < 0.001, compared to the A/D group.

To assess the effects of ganjang on the intestinal epithelial barrier, we measured the expression of two critical barrier components, MUC2 and TFF3, using real-time PCR and immunohistochemical staining. The mRNA levels of *Muc2* and *Tff3* were significantly reduced in the A/D group compared to those in the normal group and were restored after ganjang treatment (Figure 5a,b). Immunohistochemical analysis confirmed these findings, showing reduced expression of MUC2 and TFF3 in the A/D group compared to that in the normal group, similar to the mRNA levels (Figure 5c).

### 3.6. Effect of Ganjang on the Gut Microbiota in AOM/DSS-Induced CAC Mice

**Figure 6:**
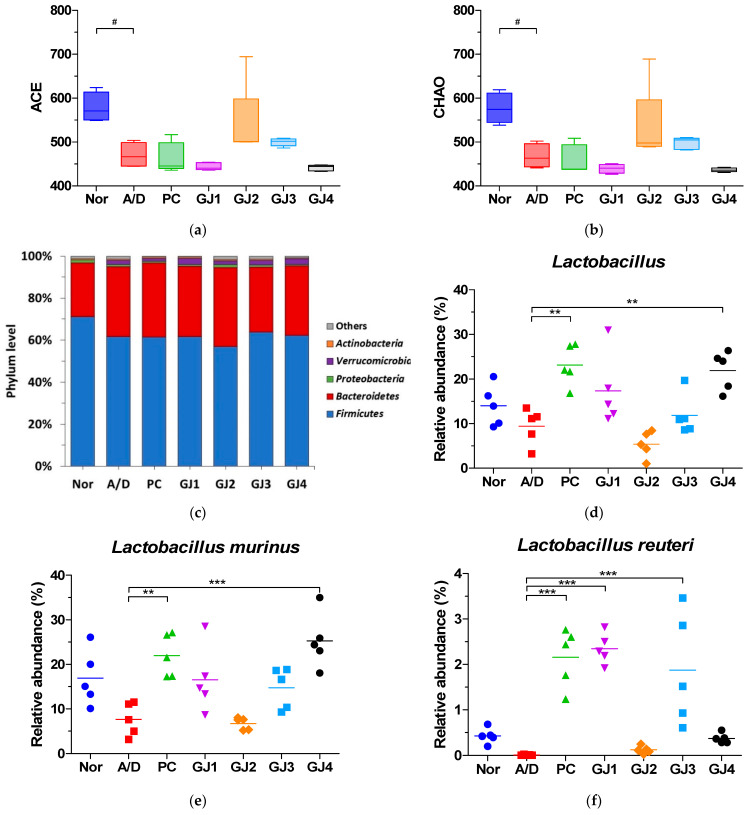
Effects of ganjang on gut microbiota diversity in AOM/DSS-induced CAC mice. Alpha diversity between the seven groups estimated using (**a**) ACE and (**b**) CHAO indices. (**c**) Microbial community composition at phylum. (**d**) genus. (**e**,**f**) species levels. Nor, Normal control group; A/D, 10 mg/kg AOM + 2% DSS; PC, 10 mg/kg AOM + 2% DSS + 75 mg/kg 5-ASA; GJ1, 10 mg/kg AOM + 2% DSS + Ganjang sample 1; GJ2, 10 mg/kg AOM + 2% DSS + Ganjang sample 2; GJ3, 10 mg/kg AOM + 2% DSS + Ganjang sample 3; GJ4, 10 mg/kg AOM + 2% DSS + Ganjang sample 4. Data are expressed as mean ± SEM (n = 5). # *p* < 0.05, compared to the Nor group; ** *p* < 0.01, and *** *p* < 0.001, compared to the A/D group.

An investigation of the effects of ganjang on gut microbiota diversity in AOM/DSS-induced CAC mice using fecal samples revealed a significant decrease in the diversity of the gut microbiota in the A/D group compared to that in the normal group (Figure 6a,b). Furthermore, NGS analysis using 16S rRNA gene amplicon sequences from the V3–V4 region in genomic DNA of six groups revealed the dominance of *Firmicutes* and *Bacteroidetes* phyla (Figure 6c). At the genus level, the relative abundance of *Lactobacillus* was lower in the A/D group than in the Nor group. Nevertheless, this reduction was recovered in the GJ groups (Figure 6d). At the species level, the abundance of *Lactobacillus murinus* and *Lactobacillus reuteri* decreased in the A/D group compared to those in the normal group, which was also recovered following ganjang administration in the GJ groups (Figure 6e,f).

## 4. Discussion

Ganjang, a traditional Korean fermented soy sauce prepared from meju (soybean bricks), is a staple ingredient in various foods [9]. As traditional ganjang is fermented through natural microbial activity, its bacterial composition can vary by region [16,17]. In the present study, we compared the protective effects of traditional and industrially manufactured ganjang against colorectal cancer (CRC).

Traditionally manufactured ganjang samples were collected from Hadong-gun, Gurye-gun, and Cheonngwon-gun, whereas industrial samples were obtained from Sunchang-gun. High-salt fermented foods such as ganjang harbor beneficial bacteria [19]. Bacterial composition analysis revealed that all the samples were dominated by *Firmicutes* and *Bacilli* at the phylum and class levels, respectively. However, the bacterial communities were more diverse at finer taxonomic levels such as class, order, family, genus, and species (Figure 1c–f). Species such as *Tetragenococcus halophilus*, *Bacillus subtilis*, *Bacillus licheniformis*, *Lactobacillus rennini*, and *Weissella confusa* were observed in ganjang samples at the species level. Differences in bacterial distribution ratios suggest that regional environmental factors may influence microbial diversity in ganjang. Studies have shown that ganjang harbors dominant bacterial species such as *Tetragenococcus* and *Bacillus*, which are essential for fermentation processes and contribute to flavor development [17]. These findings are consistent with our results, demonstrating the role of fermentation-associated bacteria in the functional and sensory properties of ganjang.

Persistent inflammation contributes significantly to cancer progression, including CRC, with chronic gut inflammation being linked to inflammatory bowel disease and CRC [20,21]. Using an AOM/DSS-induced CAC mouse model, we evaluated the protective effects of ganjang. These models exhibit typical colitis symptoms, including transient weight loss, shortened colon length, spleen enlargement, and histological abnormalities in colorectal tissues. These changes, evident in the A/D group, were mitigated by administering ganjang samples. Histological improvements included reduced crypt destruction, atypical architecture, and inflammatory cell infiltration.

Probiotics can modulate proliferation and apoptosis in cancer cells [22,23]. We measured tumor number and size to determine whether ganjang influences these processes; both were elevated in the A/D group, but were significantly reduced following ganjang treatment. An examination of apoptosis-related markers revealed that pro-apoptotic markers (*p53* and *Bax*) were decreased, whereas anti-apoptotic markers (Bcl-2 and Bcl-X_L_*)* were significantly elevated in the A/D group compared to those in the normal controls. These trends were reversed by ganjang administration. Similarly, the expression of the proliferation marker Ki-67, which was elevated in the A/D group, was significantly reduced by ganjang treatment. These findings indicate that ganjang protects against CRC by mitigating colitis symptoms and restoring the balance between proliferation and apoptosis in CAC mice.

Inflammatory cytokines, including TNF-α, IL-1β, and IL-6, play critical roles in the progression of AOM/DSS-induced CAC [24,25]. These cytokines promote cancer through pathways such as the Wnt/β-catenin signaling pathway [26]. Additionally, IL-1β is involved in cytokine production, cancer metastasis, and angiogenesis, and its overexpression is associated with poor outcomes in colorectal, lung, breast, and head and neck cancers [27,28]. Therefore, we investigated the effects of ganjang on inflammatory cytokines. Elevated levels of inflammatory cytokines, including TNF-α, IL-1β, and IL-6, were observed in the A/D group compared to those in the normal group at both the mRNA and protein levels, which were subsequently decreased by the administration of ganjang samples. These results suggest that ganjang exerts protective effects against CRC by suppressing inflammatory cytokines, including TNF-α, IL-1β, and IL-6, in AOM/DSS-induced CAC mice.

The intestinal epithelial barrier is crucial for preventing infections and maintaining intestinal homeostasis [29,30]. Key components of this barrier, such as MUC2 and TFF3, are essential for normal intestinal function [31]. A previous study showed that the expression of MUC2 and TFF3 is decreased in patients with colorectal cancer; therefore, MUC2 and TFF3 are used as diagnostic markers for colorectal cancer [32,33]. The MUC2 and TFF3 mRNA and protein levels in the A/D group were lower than those in the normal group. These reductions were reversed by the administration of ganjang samples. These findings highlight the protective role of ganjang in restoring intestinal barrier function in CAC mice.

Gut microbiota composition is strongly associated with CRC [34,35]. Studies have demonstrated that patients with colorectal cancer exhibit reduced microbial diversity [34]. Fermented foods containing probiotics alter gut microbiota diversity [35]. Consistent with previous studies, the present study showed that ganjang samples contained various fermentation-associated bacteria, including *Tetragenococcus halophilus*, *Bacillus subtilis*, *Bacillus licheniformis*, *Lactobacillus rennini*, and *Weissella confusa* [36,37,38,39,40]. In this study, an analysis of fecal samples revealed that the decreased gut microbiota diversity in the A/D group was restored by ganjang administration (Figure 6a,b). Specifically, the relative abundance of bacterial species, including *Lactobacillus murinus* and *Lactobacillus reuteri,* increased following the administration of ganjang samples. These results indicate that ganjang protects against CRC by enhancing the diversity of the gut microbiota.

## 5. Conclusions

This study investigated the bacterial composition of ganjang samples manufactured in various regions of Korea as well as their protective effects against CAC. Ganjang samples enriched with fermented-food-associated bacteria ameliorated CAC symptoms, including reduced colon length, enlarged spleen size, histological abnormalities, tumor size, and tumor number. These protective effects were mediated by altering the expression of inflammatory cytokines, regulating cell proliferation and apoptosis, and restoring intestinal epithelial barrier components. Furthermore, ganjang administration enhanced gut microbiota diversity by increasing the abundance of beneficial bacteria. Collectively, these findings suggest the potential of ganjang as a functional fermented food for the prevention and management of CRC.

## Figures and Tables

**Table 1:** Information on Ganjang samples.

	Production Method	Production Region	Water Content (%)	pH	Nutrition Facts		
					Calories (kcal/100 g)	Fat (g/100 g)	Sodium (mg/100 g)
Ganjang 1	Traditional	Hadong-gun, Gyeongsangnam-do	64.06	5.73 ± 0.01	29.87	0.19	6.49
Ganjang 2	Traditional	Gurye-gun, Jeollanam-do	68.13	5.19 ± 0.01	48.00	0.18	7.81
Ganjang 3	Traditional	Cheongwon-gun, Chungcheongbuk-do	71.43	4.48 ± 0.01	30.59	0.11	7.89
Ganjang 4	Industrial	Sunchang-gun, Jeollabuk-do	69.60	4.77 ± 0.01	51.70	0.10	7.73

**Table 2:** Primer sequences.

Gene	Forward (5′-3′)	Reverse (5′-3′)
*p53*	CCCCTGTCATCTTTTGTCCCT	AGCTGGCAGAATAGCTTATTGAG
*Bax*	AGACAGGGGCCTTTTTGCTAC	AATTCGCCGGAGACACTCG
*Bcl-2*	GCTACCGTCGTGACTTCGC	CCCCACCGAACTCAAAGAAGG
*Bcl-X_L_*	GGCACTGTGCGTGGAAAGCGTA	CCGCCGTTCTCCTGGATCCA
*TNF-α*	CTGAACTTCGGGGTGATCGG	GGCTTGTCACTCGAATTTTGAGA
*IL-1β*	CAACCAACAAGTGATATTCTCCATG	GATCCACACTCTCCAGCTGCA
*IL-6*	TGTCTATACCACTTCACAAGTCGGAG	GCACAACTCTTTTCTCATTTCCAC
*MUC-2*	ATGCCCACCTCCTCAAAGAC	GTAGTTTCCGTTGGAACAGTGAA
*TFF-3*	TAATGCTGTTGGTGGTCCTG	CAGCCACGGTTGTTACACTG
*β-actin*	CGGTTCCGATGCCCTGAGGCTCTT	CGTCACACTTCATGATGGAATTGA

## Data Availability

The data presented in this study are available upon request.
